# Reassessing the success of experts and nonexperts at correctly differentiating between closely related species from camera trap images: A reply to Gooliaff and Hodges

**DOI:** 10.1002/ece3.5255

**Published:** 2019-05-20

**Authors:** Daniel H. Thornton, Travis W. King, Arthur Scully, Dennis Murray

**Affiliations:** ^1^ School of the Environment Washington State University Pullman Washington; ^2^ Department of Biology Trent University Peterborough Ontario Canada

## Abstract

We present a reply to a recent article in Ecology and Evolution (“Measuring agreement among experts in classifying camera images of similar species” by Gooliaff and Hodges) that demonstrated a lack of consistency in expert‐based classification of images of similar‐looking species. We disagree with several conclusions from the study, and show that with some training, and use of multiple images that is becoming standard practice in camera‐trapping studies, even nonexperts can identify similar sympatric species with high consistency.

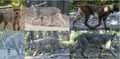

In a recent issue of *Ecology and Evolution*, Gooliaff and Hodges (2018) presented the results of a study that measured agreement in classifying images of similar species from photographic records (“Measuring agreement among experts in classifying camera images of similar species,” Ecology and Evolution https://doi.org/10.1002/ece3.4567). Gooliaff and Hodges used an example of classifying images of Canada lynx (*Lynx canadensis*) and bobcat (*Lynx rufus*), which are similar mid‐sized felids that are sympatric in several areas of the United States and Canada. The authors presented single images (taken from a variety sources) of these two species to 27 different experts and had them classify the images as “lynx,” “bobcat,” or “unknown.” Although the true identity of the species was not known in most cases, Gooliaff and Hodges assessed agreement in species classification and found surprisingly low levels of agreement between experts; to some degree, agreement was impacted by factors such as season, location, and time of day of photographic capture. Consequently, Gooliaff and Hodges suggest that studies based on classification of photographic images such as those from camera‐trapping may be unreliable when co‐occurring species are difficult to differentiate. Gooliaff and Hodges conclude that there is a need to assess the accuracy of species identifications from photographs, but we consider that several points of inference require additional scrutiny. First, the Gooliaff and Hodges's study is not broadly representative of wildlife camera‐trapping studies because it used single images to portray a capture, whereas many contemporary camera studies obtain multiple images and variable angles per capture. Second, Gooliaff and Hodges claim that their assessment represents a best‐case example of agreement because of the use of high‐quality photographs in their analysis, yet, as we show below, the use of single images may have made their study a worse‐case example of agreement. Third, they advise that multiple experts (five is suggested) should be consulted on the identification of images of similar sympatric species, but this number is impractical to apply and questionable based on their study design. Fourth, they conclude that misclassification rates would be even higher when classified by nonexperts despite not having tested this assertion explicitly. Given that Gooliaff and Hodges's study could call into question information from the many camera‐trapping studies that are being used to assess species distribution, we sought to re‐assess the veracity of their conclusions.

We conducted an image‐based species identification study using similar methodology to Gooliaff and Hodges but with undergraduate students (i.e., nonexpert observers, hereafter “NEOs”) performing the classification; Gooliaff and Hodges used experts that had previous experience (field or image identification) with the species, including agency biologists, academics, and consultants. We presented 56 NEOs with a series of 40 photographic sets (20 lynx and 20 bobcat) to classify, which had previously been identified to species level by the first author and two graduate students with substantial experience in image identification of the two species. As pointed out by Gooliaff and Hodges, bobcat and lynx share common physical characteristics but several features like the tail, paw, and leg morphology and coloration characteristics are both distinctive (Hunter, 2011; Lariviére & Walton, 1997; Koehler & Aubry, 1994; Sunquist & Sunquist, 2002) and easily discerned from most camera‐trapping images (Figure 1). We tested the ability of NEOs to classify 30 photographic sets that we determined a priori as being “standard quality” (images that are typical for camera‐trapping work, with minimal to intermediate blurring, and limited vegetation obstruction), using a random number generator to select a sequence of images from all available bobcat and lynx images from our studies in northern Washington, where the two species are sympatric (*n* = 530 total image bursts). We also used 10 randomly selected photographic sets considered as “low quality” because they had considerable blurring or distance of the image from the camera. Low‐quality images accounted for 20% of the total number of image bursts. Photographic sets consisted of between three and five images taken in rapid succession by camera traps placed along roads and trails for carnivore studies in northcentral and northeastern Washington (Scully, Fisher, Miller, & Thornton, 2018, King, unpublished data). Only 5 of the 56 NEOs had any previous experience in camera‐trapping or camera‐based image identification, and none had prior field experience with lynx or bobcats. NEOs received a brief lecture (15 min) on how to distinguish the species in photographs (e.g., noting that for lynx, the tail is short with a completely black tip and that for bobcat, the tail is longer and black only on the top half; Sunquist & Sunquist, 2002; Hunter, 2011), and were provided a booklet to refer to during identification that showed the key distinguishing features of the species. After training, the NEOs worked completely independently to identify the 40 photographic sets as bobcat, lynx, or unknown. We assessed agreement among NEOs using Fleiss' Kappa, as this was used by Gooliaff and Hodges. Note that we only used images collected during summer, with no contextual or location information, with all images presented against a forest background. According to these conditions, our images should have been among the hardest to differentiate based on the results of the Gooliaff and Hodges's study (see their table 3).

**Figure 1:**
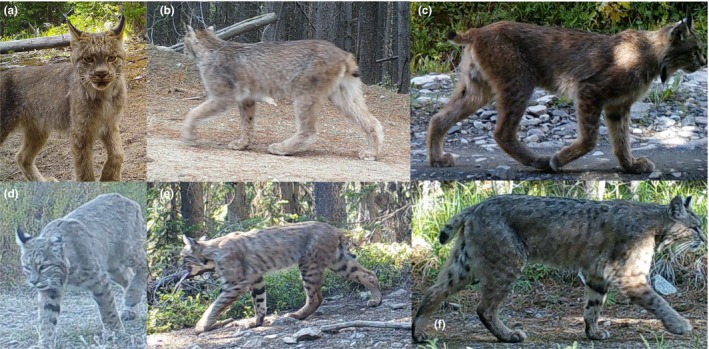
Images of lynx (a,b,c) and bobcat (d,e,f) obtained from camera‐trapping in Washington. (a and d) shows a frontal view that lacks many of the distinguishing features of the two species, making an identification to species more difficult. However, (b,c,e, and f) shows a whole‐body side view, which includes distinguishing features that are often visible in photographs from camera traps, including the short, solid‐black tip tail of the lynx, versus a longer tail with white underside in the bobcat, the larger paws and long legs of lynx compared to bobcat, and the relatively greater spotting of the bobcat. These types of whole‐body side views are common in camera‐trapping studies that often set cameras perpendicular to roads or trails. We note that for the individuals present in (a and d), we obtained subsequent side views that allowed easy identification.

For the 30 standard quality photographic sets, we found a high level of agreement among NEOs (*K* = 0.89, 95% CI = 0.84–0.95), which was only slightly reduced when we included the 10 additional low‐quality data sets (*K* = 0.87, 95% CI = 0.83–0.93). Overall, out of 2,240 classifications by NEOs, 2,163 (97%) were in agreement with our expert classification, and no NEOs used the “unknown” category. The high level of agreement among nonexperts in our study contrasts starkly with the lower levels of agreement among experts in Gooliaff and Hodges study, where overall agreement as assessed by Fleiss's Kappa was only equal to 0.64 (95% CI = 0.60–0.68), and was even lower when based exclusively on summer images (0.36, 95% CI = 0.21–0.52).

Why the large discrepancy between our study and Gooliaff and Hodges's study, particularly when the NEOs in our study had lower levels of baseline knowledge of these two species? We suggest two reasons that may explain these markedly different results and suggest that these may have relevance more broadly to wildlife camera‐trapping research. First, we provided a brief training on the morphological and coloration differences between the two species prior to the identification process. This training consisted of a brief PowerPoint lecture, highlighting the physical differences of the species that can be seen often in camera‐trapping photographs, including tail‐tip length and coloration, paw size, leg length, and coat spotting (Figure 1). During the training, we highlighted the need to take advantage of the full photographic series for assessing these distinguishing features. Our goal for the training was to ensure that all participants knew the distinctive characteristics of the species that have been pointed out in previous literature (Hunter, 2011; Lariviére & Walton, 1997; Koehler & Aubry, 1994; Sunquist & Sunquist, 2002). In contrast, Gooliaff and Hodges did not provide a priori training or initial assessment of baseline knowledge for distinguishing the two species, but instead they selected as experts individuals who had prior field or image classification experience with either species. They even included individuals who had only ever worked on one of the two species. Because of this limitation, we question whether all experts were sufficiently aware of distinguishing features of lynx and bobcat from images. For example, a researcher that had substantial field experience with bobcats, but had never worked with lynx or needed to distinguish between the two species, would be considered an expert according to Gooliaff and Hodges's criteria. This situation could have contributed to a lack of agreement among experts. Moreover, field experience with one or both species also may not equate to knowing the distinguishing features in images. For example, one of the sample images presented in the Gooliaff and Hodges article (see their table 2, image F) shows an animal having an obvious white underside to the tail, which is a distinguishing feature of a bobcat tail (Hunter, 2011; Sunquist & Sunquist, 2002); 9 experts identified this animal a lynx. This suggests that some experts did not have knowledge of tail features that distinguish the two species, likely because they never had to distinguish the two species in photographs during their previous work. Accordingly, we infer that focused training in photographic identification and species differentiation is crucial in studies involving similar species. The importance of training for data quality and consistency among nonexpert volunteers and citizen scientists is well‐recognized (Crall et al., 2011; Newman, Beusching, & Macdonald, 2003) and surely is comparably important when differentiating similar species, even among trained professionals.

Secondly, the nature of the images presented for classification could have influenced the contrast between our results and those of Gooliaff and Hodges. We presented multiple images of each individual (i.e., a single “photographic burst” that was obtained from the camera trap). We note that recent features on virtually all trail camera models include a burst of images when the camera is triggered, and the resulting series of images allows different views of individuals which may improve classification (Rovero, Zimmermann, Berzi, & Meek, 2013). Increasingly, burst or video settings (which would also provide multiple image angles) are being used in camera‐trapping studies and are quickly becoming standard practice among researchers (Comer et al., 2018; Davis et al., 2018; Hedwig et al., 2018; Ladle, Steenweg, Shepherd, & Boyce, 2018; McCarthy et al., 2018). Likewise, Gooliaff and Hodges note that the use of multiple images may result in an improved classification, but this added realism was not included in their analysis. Although not all camera‐trapping studies generate a burst of images, we suggest that the use of multiple photographs is highly important and should be encouraged, when conducting camera‐trapping studies on similar sympatrics. The side, whole‐body view obtained from camera trap images may also contribute to more accurate classifications, and this kind of view is the common result of camera‐trapping studies, where camera traps are often placed perpendicular to roads or trails. For example, based on the entire set of 530 image burst of lynx and bobcats that we have collected in Washington, less than 3% do not contain a side view. A stand‐along single image of the front of the body, face of an individual, or an animal that is sitting (which were included in Gooliaff and Hodges; see figure 4 in their paper) lack the identifying features of the side views (such as the short, all black‐tipped tail; see our Figure 1 and Appendix 1). Therefore, the claim by Gooliaff and Hodges that the high‐quality (e.g., limited blurring) images used in their study would be easier to classify than those typically collected from camera‐trapping studies and thus represent a best‐case scenario of agreement is not well supported. Although our work speaks to identification of lynx and bobcat from photographs, we note that our conclusions may apply to other similar species mentioned in Gooliaff and Hodges' article, such as mule deer (*Odocoileus hemionus*) and white‐tailed deer (*Odocoileus virginianus*), which may be hard to identify from certain angles (front) or when there is only part of an image, but quite easy from others such as whole‐body side images (e.g., the two species have very distinctive tails; Appendix 2). Another example is with black bear (*Ursus americanus*) and grizzly bear (*Ursus arctos horribilis*), where multiple images and angles allow for identification of one or more distinctive characteristics (such as claw length, ear, and snout shape) that could be hard to see from a single image (Ladle et al., 2018 for an example of a camera trap study that uses photographic bursts and distinguished the two bear species).

In the absence of hybridization that could potentially mix morphological characteristics of lynx and bobcat (which is absent from genetic studies conducted in the western United States; Koen, Bowman, Lalor, & Wilson, 2014), it seems unreasonable to assert that standard camera‐trapping images (Figure 1 b, c, e, and f and Appendix 1) would not be classified correctly by observers who were aware of distinguishing features between the two similar species, particularly when multiple images and angles of view are available. Indeed, our study with NOEs suggests that they can consistently identify such photographs in agreement with other NOEs and with our expert‐based identifications. Given those results, and the use of single images and lack of knowledge assessment/training of experts by Gooliaff and Hodges, we find their claim that 5 experts must be consulted when identifying images of similar sympatrics to be questionable. This is significant, as following such a rule in any given study would put an undue burden on researchers and the experts that they would need to consult, when modern day camera studies can generate thousands of images of focal species.

We conclude that photographic records of similar species can be hard to distinguish if they are presented singly, at angles that lack identifying characteristics, or presented to individuals that may lack knowledge of distinguishing features in photographs. However, with proper training or standardization in the features to look for, and use of multiple images obtained from standard camera‐trapping protocols, similar species can normally be distinguished with high consistency even when image classification involves nonexperts. This is good news, considering the massive amount of camera‐trapping data being collected all over the world (Steenweg et al., 2017), the existence of many similar‐looking sympatric species, and the increasing use of volunteers and citizen scientists to identify images. Finally, we are in agreement with Gooliaff and Hodges that classification of similar species should receive more attention, and perhaps the reliability, or at the very least the procedures used to classify similar species, be reported along with other metadata as common practice in camera‐trapping studies.

## AUTHOR CONTRIBUTIONS

All authors contributed to the conception and design of the work, or the collection, analysis, and interpretation of data. All coauthors assisted in drafting or editing the manuscript.

## Data Availability

Supporting information associated with this article (example photographic sets, NEO training manual, and raw results of the NEO species identification exercise) is provided on Dryad.
