# Supplementary material for: Reassessing the success of experts and nonexperts at correctly differentiating between closely related species from camera trap images: A reply to Gooliaff and Hodges
Source: Ecol Evol. 2019 May 20;9(11):6172–5. doi: 10.1002/ece3.5255 (PMC6580297; doi:10.1002/ece3.5255)

Appendix 1: Sequence of 3 images for lynx (top) and bobcat (bottom). Note that identification of these species from the first images in the burst that present incomplete side views would be difficult, but latter images clearly show several distinguishing features, including the short tail with all black tip, reduced spotting, and long legs and sloping profile of the lynx.
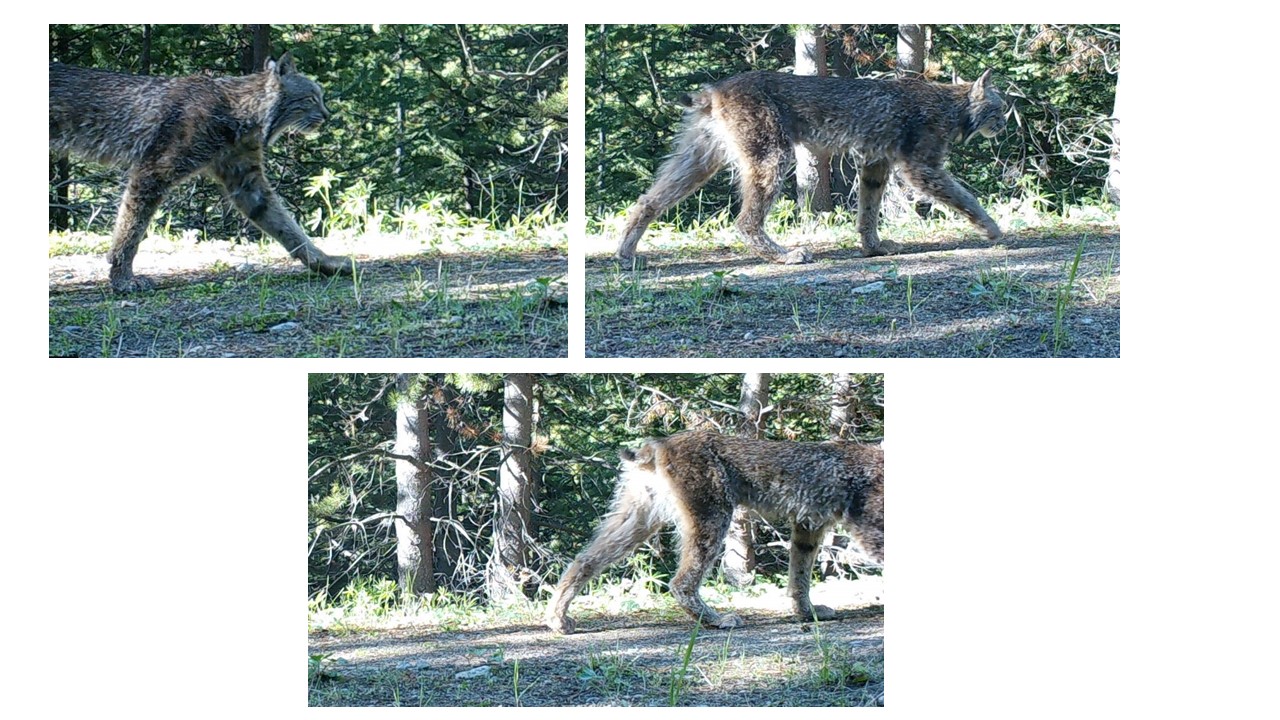

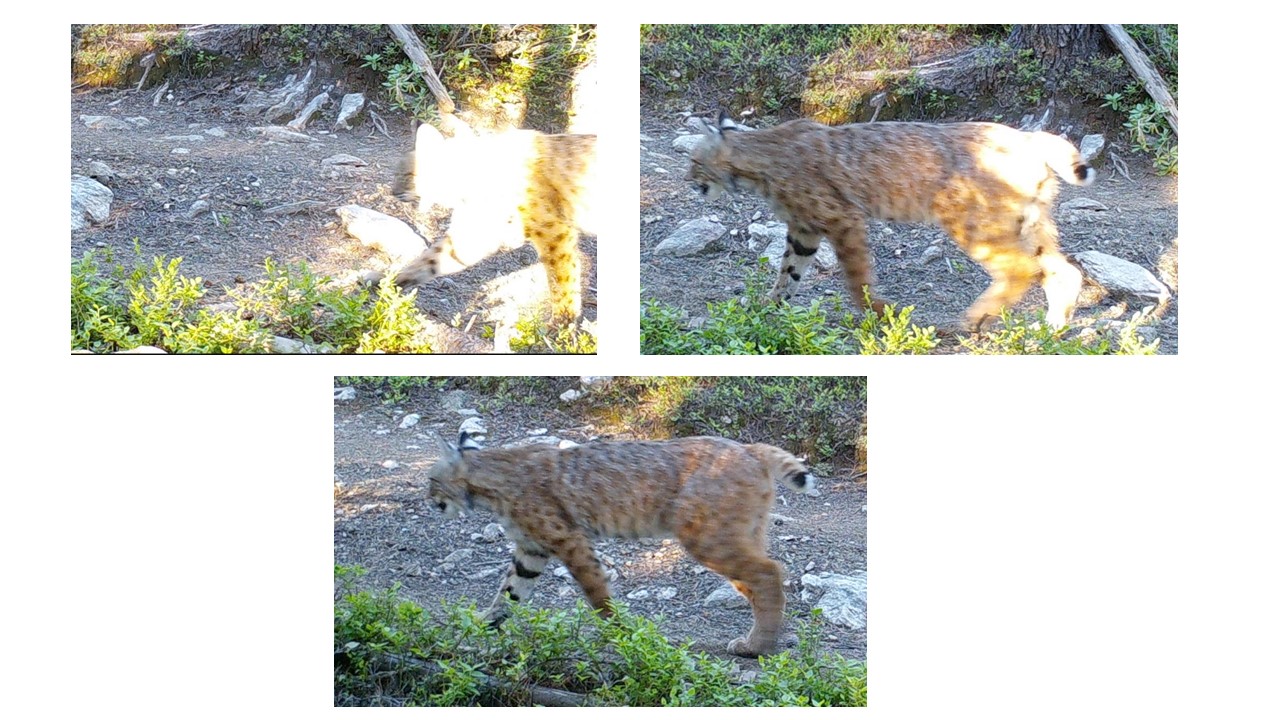


Appendix 2: Sequence of images of mule deer (top) and white-tailed deer (bottom). Although these present a nice side view of the two species, note that the first couple of pictures do not provide images of the tail, which have very characteristic differences (large black tail tip for mule deer). However, with a full image burst, views of the tails are present in latter images, allowing easy identification of these two similar species.
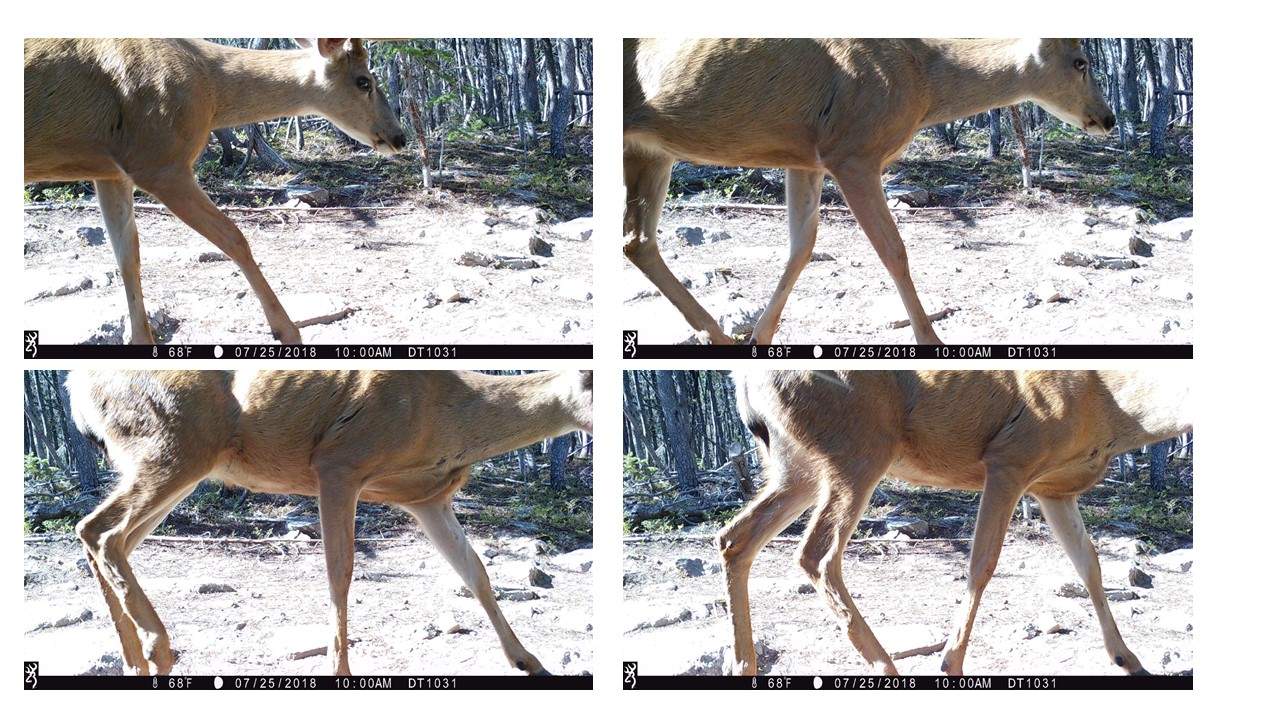

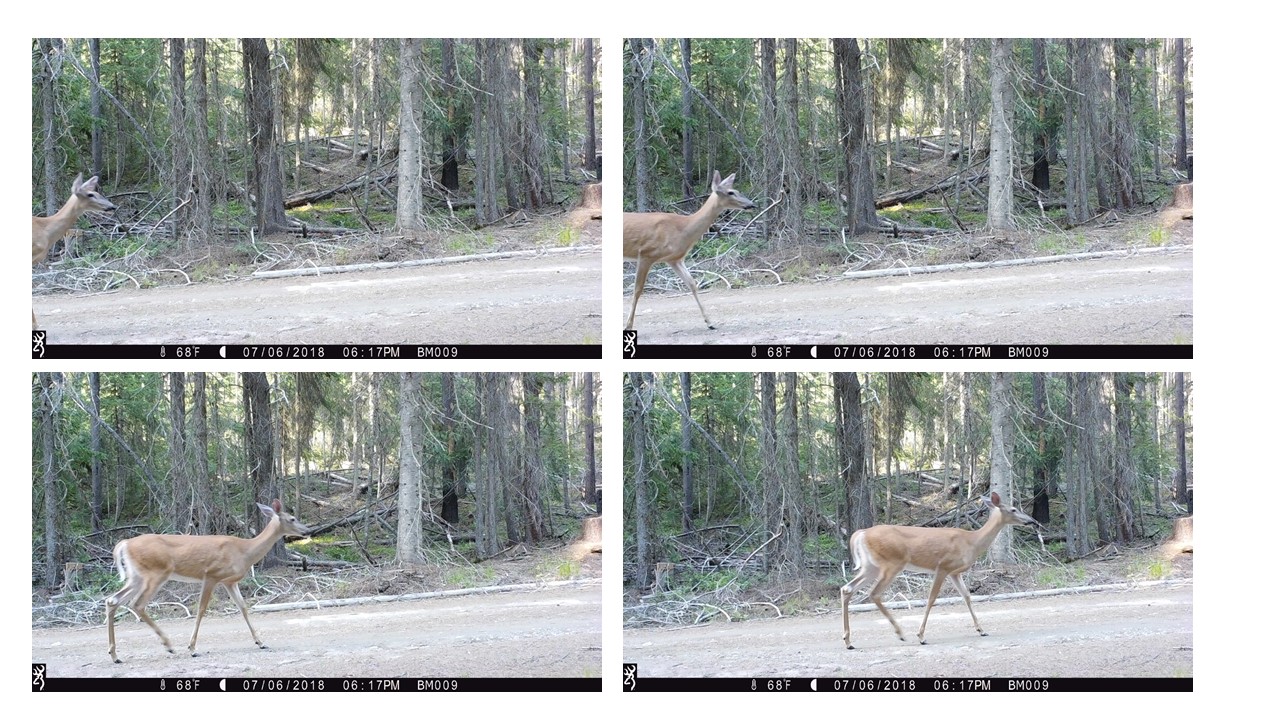

Supplement: Supplementary file 1 [file ECE3-9-6172-s001.docx]
